# Global consequences of the war in Ukraine: the last straw for (liberal) interventionism?

**DOI:** 10.1007/s42597-022-00089-1

**Published:** 2023-01-11

**Authors:** Anna Geis, Ursula Schröder

**Affiliations:** 1 Helmut-Schmidt-Universität/Universität der Bundeswehr Hamburg, Hamburg, Germany; 2 Institut für Friedensforschung und Sicherheitspolitik (IFSH), Universität Hamburg, Hamburg, Germany

**Keywords:** Liberal interventionism, Peacekeeping, Peacebuilding, Russia, Mali, United Nations Security Council, Liberaler Interventionismus, Peacekeeping, Peacebuilding, Russland, Mali, Sicherheitsrat der Vereinten Nationen

## Abstract

The interpretation of the Russian war of aggression against Ukraine as a “turning point” indicates that long-established parameters of securing peace in international politics will change. This article tentatively assesses consequences of this war for the agenda of international peacebuilding and for wider interventionist practices in contemporary violent conflict settings. It is argued that the ongoing war speeds up a preexisting phase of profound order transformation that will considerably change the ways in which states manage conflicts and attempt to secure lasting peace in the international system. The article outlines how ongoing transformations already affect—and will further affect—(liberal) interventionism at different entangled scales, ranging from the global to the local. The contribution draws on recent studies of the rise and decline of global orders and interweaves them with insights from empirical peace and conflict research and critical studies of peacebuilding and interventions. It discusses the further decline of global cooperation in the wake of the Russian war against Ukraine, outlines the consequences of the rivalry between United Nations Security Council veto powers for the global agenda of peacebuilding and the conduct of peace operations, and illustrates the local consequences for specific intervention sites, in particular the negative impact of the employment of the Russian *Wagner Group *in Mali.

## Abstract (de)

Die Interpretation des russischen Angriffskrieges gegen die Ukraine als „Wendepunkt“ deutet darauf hin, dass sich die seit langem etablierten Parameter der Friedenssicherung in der internationalen Politik verändern werden. In diesem Artikel werden einige Folgen dieses Krieges für die Agenda des internationalen Peacebuilding und für weitergehende Interventionspraktiken in zeitgenössischen Gewaltkonflikten vorläufig bewertet. Es wird argumentiert, dass der andauernde Krieg eine bereits bestehende Phase eines tiefgreifenden Ordnungswandels beschleunigt, der die Art und Weise, wie Staaten Konflikte bewältigen und versuchen, dauerhaften Frieden im internationalen System zu sichern, erheblich verändern wird. Der Artikel skizziert, wie diese Transformationen den (liberalen) Interventionismus auf verschiedenen, miteinander verflochtenen Ebenen – von der globalen bis zur lokalen Ebene – bereits beeinflussen und weiter beeinflussen werden. Der Beitrag stützt sich auf neuere Studien zum Aufstieg und Niedergang globaler Ordnungen und verwebt diese mit Erkenntnissen aus der empirischen Friedens- und Konfliktforschung und kritischen Studien zu Friedensförderung und Interventionen. Er erörtert die Verschlechterung der globalen Zusammenarbeit im Gefolge des russischen Krieges gegen die Ukraine, skizziert die Folgen der Rivalität zwischen den Vetomächten des Sicherheitsrates der Vereinten Nationen für die globale Agenda der Friedenskonsolidierung und die Durchführung von Friedenseinsätzen und veranschaulicht die lokalen Folgen für bestimmte Einsatzorte, insbesondere die negativen Auswirkungen des Einsatzes der russischen „Gruppe Wagner“ in Mali.

## Introduction

The interpretation of the Russian war of aggression against Ukraine as a “turning point” not only for the European peace and security order but also for global politics indicates that long-established parameters of securing peace in international politics will change. This contribution tentatively—and selectively—assesses consequences of this war for the agenda of international peacebuilding and for wider interventionist practices in contemporary violent conflict settings, often framed as counter-terrorism and stabilization operations. We understand these interventions as world-ordering practices, justified with references to existing or presumed norms of international order (Brock and Simon 2021, p. 4). Often referenced as “liberal peacebuilding” in academic and policy debates (see Paris 2010), this guiding concept has more recently given way to stabilization and counter-terrorism (Karlsrud 2019). (Liberal) interventionism in this paper thus refers to the collection of ordering practices that make up multilateral interventions into conflict-affected states and situations of fragility around the world. This forum contribution discusses how the war in Ukraine already alters—and will plausibly further affect—(liberal) interventionism at different entangled scales, ranging from the global to the local. We argue that the ongoing war speeds up a preexisting phase of profound order transformation that will considerably change the ways in which states manage conflicts and attempt to secure lasting peace in the international system.

To make this argument and to provide some first empirical observations, our contribution draws on recent studies of the rise and decline of global orders and interweaves them with insights from empirical peace and conflict research and critical studies of peacebuilding and interventions. In contrast to revived political narratives of an intensified geo-political rivalry between democracies and autocracies that are increasingly pitted against each other, we hold that the complexity and diversity of current and future trends in security practices cannot be grasped by such simplifying frames of interpretation (see further Higgott and Reich 2022). And even though the research literature oftentimes focuses solely on the relatively abstract level of global reordering, or, alternatively, on smaller-scale empirical assessments of transformative dynamics in individual locales, we find that it is necessary to overcome these divides. This is because we are in fact looking at processes “that are as much located at the local level as the global level, and (…) (that) are characterized by complex and interconnected processes of co-constitution between different levels of ordering” (Flockhart and Korosteleva 2022, p. 468).

This forum piece contributes to a debate that seeks to highlight both the “messiness” of current transformations and to explore first trajectories of patchworked, rival and declining forms of interventionism that are emerging today. The contribution is structured as follows: The next section (Sect. 2) deals with the enhanced rivalry among the veto powers within the United Nations Security Council (UNSC). This highlights the further demise of the “liberal” international order and the decline of global cooperation in the wake of the Russian war against Ukraine. The following section (Sect. 3) discusses consequences of this rivalry for the global agenda of peacebuilding and the conduct of peace operations. Section 4 turns to local consequences for specific intervention sites, in particular the negative impact of the employment of the Russian *Wagner Group *in Mali. A brief outlook (Sect. 5) concludes the discussion.

## Decline of global cooperation: the United Nations and “double standards” of the Western powers

The war in Ukraine fuels debates about the crisis of the international (liberal) order (Mearsheimer 2019). Today, as Flockhart and Korosteleva (2022, p. 466) argue, we are in the “final stages of the transformation of the global rules-based order into a new global ordering architecture characterized by diversity and plurality.” This notion of an international order refers to a family of increasingly contested political concepts that emerged after the end of the Second World War. As their common core, they share an understanding of a world order substantially and procedurally characterized by a varying set of global (at least in their claims) rules and norms. Chief among them are a preference for multilateral and rules-based global cooperation patterns within inclusive international institutions; for economic liberalism, open markets and free trade; as well as an expectation of the rise of liberal democratic states through democratization processes in the world (Ikenberry 2012). This intellectual hegemony of a “liberal order” has been challenged not only by processes of “democratic backsliding” (Bermeo 2016) in many parts of the world, but also by the increasing contestation of a post-Second World War set of institutions that is not perceived as inclusive by substantial parts of the Global South (Zürn 2018; Lake et al. 2021). The long-standing discontent of states in the Global South with a system of international institutions considered as unequal, non-inclusive and biased (e.g. Nel 2010) has also become visible in rifts in cooperation on shoring up international support for Ukraine (Tripathi 2022).

The Russian war against Ukraine has further exacerbated the increasing rivalry between Western powers and China and Russia within the United Nations Security Council that already manifested in the frequency and continuity of vetoes cast by Russia and partly China in the Syria crisis (Niemann 2018). The United Nations (UN) is currently seen to be in an “existential crisis” (Puri 2022, p. 8) due to Russia’s war of aggression against its neighbouring country. The veto power Russia has violated basic principles of international law by its invasion of Ukraine, *inter alia* it has breached the territorial integrity of a sovereign state and massively targets civilians in this war. The veto system of the UNSC prevents this institution from dealing appropriately with situations where one of its veto powers, possessing nuclear weapons (and indicating threats of their use), conducts a war of aggression—or where such a veto power seeks to protect another regime that is accused of a brutal war against its own civilians as in the Syrian case.

The inability of the UN Secretary-General António Guterres to mediate in the Russian war against Ukraine and of the UNSC to deal appropriately with the situation has caused a lot of frustration. The Ukrainian President Zelenskyy expressed this in his speech to the UNSC in April 2022 in light of evidence of Russian atrocities in Bucha: “How is this different from what the ISIS terrorists were doing? (…) Except that it is done by a permanent member of the United Nations Security Council. (…) The U.N. system must be reformed immediately so that the right of veto is not a right to kill.” The only logical alternative would be to concede the council’s irrelevance and “dissolve yourself altogether.”^1^

However, some observers emphasize that the UN system does have some positive effects also in *this* situation and that it should not be reduced to the UNSC’s (in)ability to pass resolutions (Puri 2022): It is the only global platform where the war is discussed on a regular basis, in the presence of Russian and Ukrainian representatives, and where Russia is exposed to heavy criticism. In addition, the initiative of a group of UNSC members to transfer consideration of the matter to the UN General Assembly is seen as progress, the result of 141 (out of 193) states condemning Russia’s war of aggression on 2 March 2022 as an overwhelming success (Nagelhus Schia 2022).^2^ This UN General Assembly vote was seen by many in the West as an important signal that “the world” (at least in its large majority) is emphatically reaffirming international norms in times of their blatant breach by a P5 member. More critical reviews, however, point out that a significant number of countries of the Global South, among them India, did abstain in this and other related votes and also do not take part in the sanctions regime against Russia. Many Global South countries see themselves in a position of not having “choices” due to their strong dependencies on powerful actors, while the wealthy and powerful states of the Global North (once again) have the privilege to make and enforce—also painful—choices (Tripathi 2022).

There are quite different motives for these countries to abstain, among them their dependency on arms sales and wheat exports from Russia and their justified concerns about their food security. However, what is especially relevant for our topic of global interventionism is the criticism that a number of governments from the Global South also voice in this situation: Western powers have often enough violated international law themselves, fought wars of aggression (Iraq 2003), conducted military interventions without a UNSC mandate (Kosovo 1999), transgressed their mandate in the Libyan case in 2011 with the aim of regime change, destabilized whole regions with their interventions (Middle East) with enduring devastating effects (Yahya 2022), and still present themselves as morally superior guardians of a “rules-based order”—while *they* make these rules and break them at *their* will. As Tripathi (2022) observes: “The argument, on the lines of postcolonial and decolonial approaches in IR, given by most countries (who have experienced worse sides of international order) is that this ‘rules-based order’ has not been rules-based in reality; instead, it has allowed the prominent powers like the US to violate international law with impunity whether it was Iraq, Afghanistan, former Yugoslavia, or others.”

Such “double standards” by the West have been stigmatized by Russia for a long time, even before President Putin came to power (Allison 2013). This manifested not only in the Russo-Georgian war (2008). It culminated in the Ukraine war when Russian governmental actors proposed similar justifications for their own use of force that have been put forward by Western actors, such as preventing genocide, protecting civilians under a duty of a responsibility to protect (here: Russian citizens only), fighting terrorism, or the pursuit of weapons of mass destruction, or aiming at regime change. By referring to the lack of credibility of Western actors, Russian governments have sought to implement a special right of their own to intervene in the so-called “post-Soviet space” or “near abroad”, to provide a Russian style of “peacekeeping” in this region, and to keep Western actors out of this space as far as possible (Allison 2013, pp. 120–169; Schaller 2018, pp. 30–35). The employment of such international law arguments by Russia in the Georgia war case can also be interpreted as “testing” international responses and seeking “tacit acceptance” of “Russian exceptionalism in its zone of ‘privileged interests’” (Allison 2013, p. 167).

The military interventions and wars in which the P5 powers have been involved since the 1990s have thus further exacerbated a basic conflict between the Western powers and Russia (and to some extent China) about who makes the rules in global politics, about their legitimation in interpreting international law, and employing the use of force. The persistent claims of Western powers to use military force for good and reasonable purposes only is definitely not shared as credible justification by others.

## (Liberal) peacebuilding: decline, rivalry, or cooperation?

While the prior section has sketched out the paralysis of the UNSC and the mutual blaming of Western Permanent Five members and Russia and China of grave breaches of international law, the following section will discuss to what extent the global agenda of (liberal) peacebuilding will plausibly be affected by the described enhanced rivalry. The liberal “peacebuilding consensus” (Richmond 2004), that is the international attempt to construct a liberal peace in conflict-affected and post-conflict states, has long been a concept and policy practice in decline, well before Russia’s invasion of Ukraine. Long (and often harshly) criticized as prone to fail, as focused primarily on international agendas to the detriment of local voices and interests, and as unaware of (often post-colonial) power dynamics at play (Campbell et al. 2011; Richmond and Mac Ginty 2015), the critique of liberal peacebuilding “has been so successful as to become the new mainstream” (Hameiri 2011, p. 192). This sustained academic critique was paralleled by a series of high-profile failures such as the multi-year interventions in Afghanistan and Mali, adding to an earlier sense of irrefutable decline and “end” (Turner and Kühn 2019, p. 238) of the liberal interventionist paradigm.

The war in Ukraine has exacerbated this pre-existing crisis of liberal interventionism, with parallel processes pointing to a further decline of liberal interventionism, at least in the field of UN peacekeeping, as well as to patterns of rival ordering attempts, but also new forms of cooperation.

As to the first process, peace operations deployed by the United Nations have been in decline since 2014, with the last new UN peacekeeping operation launched in 2014. This decline in relevance of multilateral peace operations is likely to continue in a stalled UN Security Council. In the near future, observers do not expect the UN to be “mandated to launch robust, large, and costly new UN peacekeeping operations or reform processes” (de Coning 2021, p. 216; Osland and Peter 2021). Already challenged by budget cuts in the wake of the US Trump administration and burdened by the COVID-19-pandemic, UN peacekeeping is likely to face further cuts, as political priorities and attention across Europe and beyond shift to territorial defense and within-alliance cooperation.

However, while the scope and scale of multilateral peace interventions is expected to further contract (de Coning 2021), we expect them to continue to exist and evolve further, rather than go extinct (Coleman and Williams 2021)—even in times of war and resulting conflict between major great powers in the UN Security Council. The current phase of retrenchment might conceivably lead to deployments of smaller missions with more focused goals that deviate clearly from previous instances of mission creep and resulting “Christmas tree” mandates (Oksamytna and Lundgren 2021, p. 227) that are impossible to fulfil from the start. At the same time, alternative approaches to peacebuilding have become commonplace in world politics and increasingly rival established multilateral practices. Recent research in particular highlights the increasing presence of non-Western powers as mediators and peacebuilders. As Peter and Rice (2022) show, non-Western peacebuilding practices are frequently based on unilateral interventions with a preference for bilateral forms of assistance. At the same time, many—but not all—non-Western actors favour top-down government-to-government cooperation, such as in the case of China, who is also the second-largest contributor to the UN peacekeeping budget (Abdenur 2019, p. 57). The arrival of assertive and influential new actors on the scene of peacebuilding and conflict management heralds a new era for peace operations.

But one that will not necessarily lead to the bifurcation of interventionism into rival approaches. Instead, one result of previous research is that the dichotomous distinction often made between “Western” and “non-Western” approaches to peacebuilding is overstated, as new approaches often overlap with existing (liberal) practices and “operate both within and outside the liberal peace perspective” (Peter and Rice 2022, p. 27; see also Turner and Kühn 2019). Coming to terms with the effects of the war in Ukraine on peace interventions will thus be more complicated and interwoven than simpler concepts of multi-polarity predict. Confronted with a fraying consensus on the inside and increasingly assertive Global South-alternatives to the “peacebuilding consensus” from the outside, future peace operations will need to reimagine and redesign established practices of (multilateral) cooperation. Patterns of rivalry and competition, but also new forms of cooperation between a growing number of involved actors can be envisaged in which “islands” of (liberal) interventionism, even sometimes within a specific intervention site populated by a diversity of actors, will plausibly survive, while losing their claim to universality. In practice, we are already witnessing the rise of multi-intervention sites such as in the case of Mali, where multiple actors, including Russian private military companies, pursue divergent aims that range from military training to peacekeeping and counter-terrorism operations, as we discuss in more detail in section four below. The architecture of peace operations will have to adapt to this multiplication of actors and approaches. Calls for more “strategic political coherence” (de Coning 2019, pp. 536–537) in peace operations beyond the UN system echo this perception of a changed environment. Entering a “new era of networked peace operations” (de Coning 2019, p. 537), new forms of accommodation and cooperation between a large and diverse group of actors need to be found.

## Reshaping local intervention sites by Russian “private” military companies?

The Russian war against Ukraine might also affect specific intervention sites in the Middle East and Africa. One immediate and devastating impact affects the worldwide wheat trade and the food assistance conducted by the World Food Programme. The steep reduction of wheat exports renders very critical humanitarian situations such as in Syria even worse. But also with regard to military and political “spheres of influence” that foreign state powers such as Russia, Iran, Turkey, and the US have established in Syria (Adar et al. 2022, p. 1) and the role of “proxy war” actors such as individual foreign fighters or Private Military Companies (PMCs) in violent conflicts in the Middle East and Africa, the war in Ukraine will—depending on its duration—have effects on the ground. One consequence is that Russia redirects more military forces towards its war in Ukraine and thus decreases its presence in Syria.^3^ Turkey and Iran might use this opening “space” to further increase their political influence and interventionist practices in Syria (Adar et al. 2022). Apart from reconfigurations of state power interventions, “spaces” can also be filled by the employment of non-state actors. To illustrate this, we will focus here on Russian PMCs that have attracted a lot of attention in recent years.

The increased significance of *Western* PMCs in violent conflicts has been analyzed intensively in Peace and Conflict Studies as well as Security Studies since the 2000s (e.g. Avant 2005; Krahmann 2010). While Russian PMCs also emerged in the 1990s, the year 2014 (with the violent conflict in Eastern Ukraine and the annexation of the Crimea) marked the beginning of an enhanced development and employment of Russian PMCs, although their status in Russia is not legalized. The employment of Russian PMCs is seen as an “emulation” of Western practices (Jones et al. 2021, p. 13) and some of the motives for their use by the Russian government are similar to those that have been identified for Western governments (Marten 2019, p. 198): more flexibility in their deployment, the veiling of real losses in combat since the public is not aware of casualties among PMCs. However, there are a number of differences: *inter alia*, the PMCs are not so “private”, given their narrow affiliation with the Russian executive. While it is often claimed that groups such as the most prominent “Wagner Group” are used by the Russian government to maintain “plausible deniability” (Rácz 2020) in their conduct of “hybrid warfare”, other studies point out that the deniability is hardly plausible anymore, given growing evidence of these groups’ activities in warfare and their connections to the executive (Marten 2019, p. 187, 198). In addition, Russian PMCs are also used for pro-Russian ideological warfare and might play an increasing role in harmful cyber acts and in instigating civil unrest in the future (Jones et al. 2021; Bukkvoll and Østensen 2020, pp. 14–15). Although the data basis for research on Russian PMCs remains rather scarce in comparison to Western ones, beyond their employment in Eastern European and Central Asian countries (that are regarded as so-called “post-Soviet space”) and in Syria, there seems to have been a Russian “pivot to Africa” with regard to PMC deployments (Jones et al. 2021, p. 60). Among others, PMCs—with quite differing tasks—are active in Libya, Chad, Burundi, Central African Republic, Congo, Mozambique, Sudan, Nigeria, and Mali (Jones et al. 2021, p. 15).

The Mali case is especially interesting since it indicates that the use of Russian PMCs for military training of state armed forces also influences Western calculations about their own engagement in an intervention site: Since 2012, the violent conflict in Mali has developed into a multi-intervention site, in which the United Nations Multidimensional Integrated Stabilization Mission in Mali (MINUSMA), the French counter-terrorism operations *Serval* and *Barkhane*, the European Union (EU) Training Mission (EUTM), and the G5 Sahel Joint Force have been active. Mali exemplifies the growing entanglement of the international peacekeeping and the counter-terrorism regimes under the paradigm of “stabilization” (Moe and Geis 2020; Moe 2021): New flexible forms of collaboration include objectives such as countering non-state extremism and terrorism, stabilizing territories, and reinstalling state authority. The term “stabilization” became more prominent in the post-Cold War era, in particular within NATO and the US military doctrine. It gained a special momentum after 2001 and has also been adopted by the UN in its missions in Haiti (2004), the Democratic Republic of Congo (2010), in Mali (2013), and in the Central African Republic (2014). UN peacekeeping has become increasingly coercive—this so-called “robust turn” of UN peacekeeping indicates the decline of liberal peacebuilding (Karlsrud 2019).

The operational proximity of Russian PMCs, accused of human rights violations, to UN peacekeepers can have a negative impact on the local perception of UN troops, as is indicated for the case of the United Nations Multidimensional Integrated Stabilization Mission in the Central African Republic (Jones et al. 2021, p. 54). In addition, the increased availability of Russian PMCs enables governments in these settings to “pick and choose” their trainers for their militaries in cases where Western partners do not fulfill their expectations or where local populations have negative perceptions of Western intrusion (Bukkvoll and Østensen 2020, p. 19; Jones et al. 2021, pp. 61–64). The EU, engaged with a Training Mission in Mali, reacted very strongly when it became known that the Malian military government led by interim President Goïta, in power after a coup d’état, might collaborate with the Wagner Group, as France started to withdraw its forces. Josep Borrell, the EU’s High Representative for Foreign Affairs and Security Policy, announced the winding down of the EUTM in Mali, declaring in April 2022: “We have decided to suspend, to stop, certain formations of our training mission in Mali focused on the units of the armed forces of the Malian national guard. (…) There are not enough security guarantees from the Malian authorities over the non-interference of the well-known Wagner Group”. Borrell claimed that the “notorious Wagner group (…) is responsible for some very serious events which have led to tens of people being killed in Mali in recent times.”^4^

The reliance of the Malian government on the Russian Wagner Group and further news on the killings of civilians by these forces in autumn 2022 has in the meantime further strained the maintenance of MINUSMA. The stabilization mission has become ever more difficult and dangerous for the troop deploying countries: A number of countries, among them Sweden, the UK, and most recently Germany, have decided to withdraw from MINUSMA. In statements from officials of withdrawing countries, apart from the deplored lack of cooperation by the Malian government, negative references to the Wagner Group are made and fears are expressed that Mali will “fall” to Russian influence if Western troops leave the country.^5^ As the Mali case exemplifies: Given that Russian “private” military companies are used as instruments of state influence abroad and employed to maintain ambiguity around Russian state military involvement, their increased use in Africa and the Middle East exacerbates conflicts with Western interveners and providers of security training (Jones et al. 2021).

## Outlook

This paper argues that the Russian war of aggression against Ukraine has exacerbated a pre-existing crisis of liberal interventionism and will have wide-ranging effects on the ways states seek to establish and maintain peace in the international system. Focusing first on the decline of global cooperation in the context of the UN Security Council, second on new cooperation patterns in international peace operations, and third on the conflictive role of Russian “private” security companies in local intervention sites in Mali, the paper has unfolded several consequences of the Russian war at different entangled scales.

For the future, as one consequence of the outlined enhanced rivalry between the veto powers within the UNSC, we anticipate the further proliferation of ideas of “club governance” and ad-hoc informalized security arrangements. State groupings such as G7/8/20 or the BRICS (Brazil, Russia, India, China, South Africa) are also indicators of states “by-passing” the rigid UN system, attempting to coordinate international policies and partly develop their own procedures and self-legitimation strategies (Gronau 2015).

As a second trajectory, “democracy vs. autocracy” grand narratives have been fuelled again by the Ukraine war and could be further revived in the future. The idea of a “Concert of Democracies” that should enjoy privileges in global governance, including the self-mandating of the use of force, was discussed in liberal and neo-conservative circles in the US more than a decade ago (Geis 2013). US President Joe Biden, who conducted a “summit for democracy” in December 2021, has been reviving a narrative of global system rivalry in the wake of the Russian invasion of Ukraine. However, dividing the world up into two neat “camps” of democracies and autocracies misses the complexity and “messiness” of many of today’s conflict constellations (Abb et al. 2022).

Finally, new and emerging cooperation patterns in multi-intervention sites such as Mali point to the need to further question binary distinctions between “Western” and “non-Western” actors in interventions. Whether interventionist practices differ *fundamentally* is a question that requires more systematic research (Turner and Kühn 2019). In line with Heathershaw and Owen’s argument on the growing influence of authoritarian powers in shaping regional orders and managing conflicts, we expect the “liberal peace” to not be simply replaced by something else, but to make it necessary to study the intertwining of “liberal peace” projects with new illiberal institutions and discourses (2019, p. 270). While Peace and Conflict Studies and International Relations have produced a lot of research on Western interventionist practices since 1990, studies of the effects of “non-Western” and entangled interventions remain a desideratum.

## Footnotes

1 Zelenskyy’s statements cited in Taylor and Rupert (2022).

2 Furthermore, the war in Ukraine helped in April 2022 to implement an innovation that had been proposed by Liechtenstein for 18 years: The five permanent members of the UNSC are called to appear before the General Assembly and explain themselves whenever they exercise their veto (Nagelhus Schia 2022; Taylor and Rupert 2022). This measure was first employed on 8 June 2022, when Russia and China had to justify why they had vetoed the imposition of stronger sanctions on North Korea.

3 See https://www.nytimes.com/2022/10/19/world/middleeast/russia-syria-israel-ukraine.html.

4 All quotes taken from https://www.dw.com/en/mali-eu-to-reduce-security-training-operations/a-61441392.

5 See, for example, https://www.sueddeutsche.de/politik/mali-bundeswehr-aufklaerungsdrohne-1.5700720 from 22 November 2022 and https://www.thedefensepost.com/2022/11/15/uk-end-deployment-troops-mali/ from 15 November 2022.
